# Diversity of Mosquitoes (Diptera: Culicidae) in the Caatinga Biome, Brazil, from the Widespread to the Endemic

**DOI:** 10.3390/insects11080468

**Published:** 2020-07-24

**Authors:** David Campos Andrade, Sirlei Antunes Morais, Letícia Silva Marteis, Renata Antonaci Gama, Renato Cesar de Melo Freire, Belgrano Santiago Rekowski, Helene Mariko Ueno, Roseli La Corte

**Affiliations:** 1 Programa de Pós-Graduação em Ecologia e Conservação, Universidade Federal de Sergipe, São Cristóvão 49100-000, SE, Brazil; david.c7@hotmail.com (D.C.A.); santiago_rekowsky@hotmail.com (B.S.R.); 2 Departamento de Morfologia, Universidade Federal de Sergipe, Avenida Marechal Rondon, S/N, São Cristóvão 49100-000, SE, Brazil; sirlei.morais75@hotmail.com; 3 Colegiado de Medicina, Universidade Federal do Vale do São Francisco, Av. José de Sá Maniçoba, S/N, Centro, Petrolina 56304-917, PE, Brazil; leticia.marteis@univasf.edu.br; 4 Departamento de Microbiologia e Parasitologia, Universidade Federal do Rio Grande do Norte, Avenida Senador Salgado Filho 3000, Natal 59078-970, RN, Brazil; renataantonaci@hotmail.com; 5 Programa de Pós-Graduação em Biologia Parasitária, Universidade Federal do Rio Grande do Norte, Natal 59078-970, RN, Brazil; renatomfreire000@hotmail.com; 6 Programa de Pós-Graduação em Sustentabilidade—Escola de Artes, Ciências e Humanidades da Universidade de São Paulo. Av. Arlindo Béttio, 1000, São Paulo 03828-000, SP, Brazil; papoula@usp.br

**Keywords:** biodiversity, culicidae, semiarid, vector ecology, caatinga

## Abstract

Mosquito fauna in the northeast semiarid region of Brazil, Caatinga biome, are poorly known. Studies on the diversity are scarce and the few surveys available focus on local fauna. In order to understand the ecological pattern of mosquito’s distribution, information available from studies from 2008 to 2015 were gathered. A partitioning framework of the beta diversity, the turnover (βJTU) and nestedness (βJNE) components were used to determine dissimilarity among communities. Eighty-two morphospecies were recorded and 47 of the species were not shared between the areas. The most representative genera were *Aedes*, *Anopheles*, *Psorophora*, *Haemagogus*, *Coquillettidia*, and *Mansonia*, which all include species of medical interest. The communities had high rates of variation, and the mechanism of turnover accounted for the observed diversity pattern. Despite differences in collection methods, the observed dissimilarity may be related to the broad environmental heterogeneity of the biome, the intrinsic relationships of the species with their habitats, and the environmental degradation caused by different types of anthropogenic interference. Considering the mosquito species richness and endemicity, the hypothesis that the Caatinga harbor poor biodiversity is rejected. The spatial variation observed is of particular importance and should be taken into account for the knowledge of Caatinga biodiversity.

## 1. Introduction

The Caatinga biome is characterized by dry deciduous forests in different successional stages under high water deficits [1]. The biome covers 844,453 km^2^, approximately 11% of the territory of Brazil, and is almost entirely within in the northeast region, except for a small portion north of Minas Gerais State [2]. The semiarid climate and scarcity of water are related to a zone of interplanaltic depressions that prevent the movement of humid air masses. Another factor affecting climate is region’s geographical position in the global equatorial zone [3]. The fauna and flora are well adapted to the adverse conditions that characterize the Caatinga biome such as low rainfall throughout the year and high solar radiation [4]. 

The Caatinga suffers from different environmental threats that change or eliminate habitats. One serious problem is desertification, which promotes salinization and leads to unproductive soils [5]. Currently, 94% of the semiarid area of Brazil is considered moderately or highly susceptible to desertification and highly sensitive areas are increasing [6]. In addition to the degradation due to human activities, the expansion of sensitive areas has been accelerated by severe droughts that have affected the region in recent years [7]. Degradation and desertification threaten the under-studied biodiversity in the Caatinga - a biome of proven species endemism [8,9,10,11]. 

The importance of conserving the Caatinga biome has been recently recognized [12]. Currently, 7.7% of the Caatinga area is included in protected areas, but less than 20% of this area is categorized as integral protection, which is proportionally smaller than the protected areas of rainforest biomes in Brazil [13,14]. Caatinga is a highly impacted biome and the remaining areas do not form a large single block, but instead is distributed into many fragments of various sizes [13] which house different species assemblages. Studies on the diversity of Culicidae in the Caatinga are scarce and the few surveys available focus on local mosquito fauna [9,10,11]. Our study aims to join these studies of diversity on a large spatial scale and contribute to the understanding of the mosquito community’ structure and the patterns of biogeographic distribution of species [15].

Diversity can be assessed at a local level, between habitats, and across regional scales, known as alpha (α), beta (ß), and gamma (γ) diversity respectively [16]. Alpha diversity is related to the local richness, given by the total number of species in a given habitat. Beta diversity is the turnover of species along an environmental gradient [17]. While alpha diversity is related to local richness, beta diversity refers to variation in species composition between different locations in a region of interest. Beta diversity can be estimated by the inverse of the mean number of habitats occupied by each species in the Region. By making explicit the variation in the composition of species assemblages, it can contribute to the selection process of areas to be protected [18]. This may be particularly relevant to the Caatinga, in view of the biome fragmentation.

In this study, the spatial patterns and processes that generate the diversity (ß) of mosquitoes within the Caatinga were analyzed. A list of mosquito species from this region was compiled based on recent fauna inventories, which represented the largest sampling effort performed for the biome. Additionally, the similarity between the communities was analyzed in order to identify factors that, in addition to water scarcity, may influence the formation of species assemblages. It is difficult directly to compare the richness from different studies due to variation in their methods, sampling time, sampling strategies and goals. On the other hand, it is expected that the methods applied vary in line with the habitats available. Thus, this study aims to evaluate the hypothesis of heterogeneity, considering the fragmentation of the Caatinga domain, assuming that some of the findings may be related to methodological variation. We also report new data on DNA barcoding of seventeen species or morphotypes, that were performed in the context of “Mosquitos da Caatinga” project in an attempt to solve taxonomic issues. The sequences data were deposited in the GenBank at the National Center for Biotechnology Information (NCBI) search database.

## 2. Materials and Methods

### 2.1. Study Area and Collection Techniques

For the updated list of Caatinga mosquito species, we compiled data from surveys carried out in dry deciduous forests between 2008 and 2015. The species list included some original data extracted from the “Mosquitoes Caatinga” project database, previously published results of the project [9,10,11] and data from inventories conducted over the past decade presented in master dissertations or papers [19,20,21]. 

**Figure 1:**
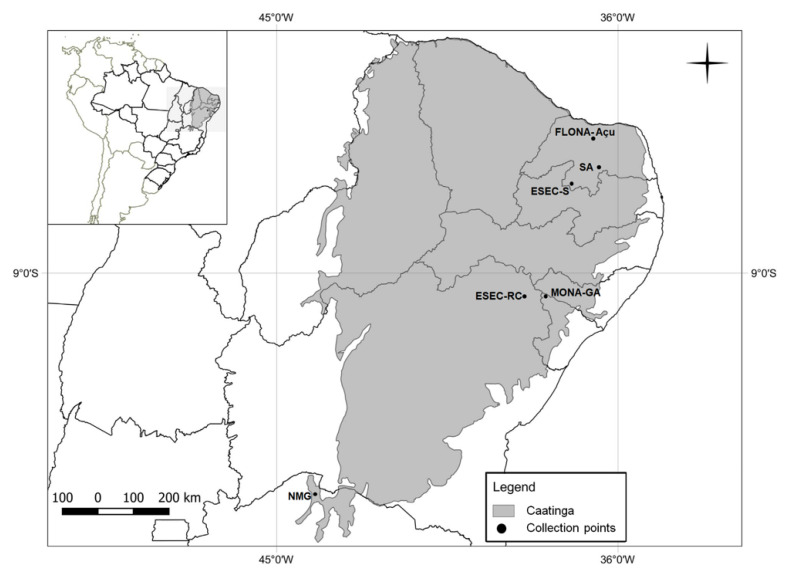
Map of the area covered by the Caatinga biome (grey shading) and the study sites in the states of Bahia (ESEC–RC, Estação Ecológica Raso da Catarina), Minas Gerais (NMG, north of Minas Gerais), Rio Grande do Norte (FLONA–Açu, Floresta Natural do Açu; ESEC–S, Estação Ecológica do Seridó and SA, Sítio Areias), and Sergipe (MONA–GA, Monumento Natural Grota do Angico) Brazil, where mosquitoes were surveyed between 2008 and 2015 (Adapted from Marteis et al., 2017 [9]).

The “Mosquitoes Caatinga” project (SISBIOTA CNPq/FAPITEC, Call 47/2010 process 563383/2010-0) surveys were conducted in the states of Sergipe, Bahia (northern), and Rio Grande do Norte. The monthly collections covered dry and rainy seasons in three protected areas between 2011 and 2014: 1. Estação Ecológica Raso da Catarina (ESEC–RC, Raso da Catarina Ecological Station) in the state of Bahia (09°33′13″–09°54′30″ S, 38°44′00″–39°29′20″ W), which covers 1000 km^2^, with rare transitory water resources and an average rainfall of 540 mm. Shrubby-arboreal forest covers most of the ecological station, characterized by a dense, spiny vegetation, dominated by Euphorbiaceae, Bromelieae and Cactaceae. Tank bromeliads, mainly *Aechmea aquilega* (Salisb.) are abundant throughout the area (data partly published in Marteis et al. [9,10]); 2. Floresta Nacional de Açu (FLONA–Açu, Açu National Forest) in the state of Rio Grande do Norte (05°34′59.4″ S, 36°56′39.3″ W), which covers 2.25 km^2^, with average rainfall of 600 mm and tree–shrub vegetation. Flona-Açu has a narrow and elongated aspect and ends in a pond that dries up in years of severe droughts (data recovered from the data merged in Inácio et al. [22]); and 3. Monumento Natural Grota do Angico (MONA–GA, Grota do Angico Natural Monument) in the state of Sergipe (09°39′50″ S, 37°40′57″ W), which covers 21.83 km^2^, with average precipitation of 550 mm, a tree–shrub forest with a predominance of *Poincianella pyramidalis* (Tul.) L. P. Queiroz (common name Catingueira). Few clusters of tank bromeliads, mainly *Hohenbergia catingae* Ule are scattered over the area (data partly published in La Corte et al. [11]). Ephemeral ponds are formed in rainy season. (Figure 1).

Overall, the collection techniques included ovitraps [23], larvitraps [24], water suction from natural containers, and entomological dippers in flooded areas to collect larvae. To capture adult mosquitoes during the day and at night, CDC miniature light traps, Shannon traps, and human landing catches were used, with the last two requiring a hand-held or battery-operated aspirator [25]. With this set of techniques, a wide variety of habitats were sampled, including phytotelmata, such as hollow trees, ground and epiphytic bromeliads, and holes in rocks and ponds (Table 1). All ponds surveyed were retention ponds, commonly excavated in northeastern Brazil to retain and store rainwater.

The information on Minas Gerais State (NMG) was published by Santos et al. [20] for an area of the Caatinga near the transition with the Cerrado (savannah) biome, which typically has semideciduous vegetation and higher rainfall, with an annual average of 871 mm. Mosquitoes were sampled in the following four protected areas in Caatinga patches in the middle portion of the São Francisco River: 1. Parque Estadual Mata Seca (Mata Seca State Park) (14°48′36″ S, 43°55′12″ W); 2. Parque Estadual Lagoa do Cajueiro (Cajueiro Pond State Park) (14°55′08″ S, 43°56′23″ W); 3. Reserva Biológica Jaíba (Jaíba Biological Reserve) (15°03′57.81″ S, 43°45′45.03″ W); and 4. Reserva Biológica Mata Azul (Blue Forest Biological Reserve) (15°11′32.20″ S, 43°54′41.10″ W). These conservations units are within an approximate perimeter of 150 km and harbor marginal ponds of the São Francisco river. The authors presented all the data pooled. Mosquitoes in flight mosquitoes during the day and at night were captured using Shannon light traps, CDC light traps, and human landing catches with hand-held aspirators. The collections occurred biannually, timed to include both the dry and rainy seasons, from August 2008 to July 2012. 

The other survey included in this study was conducted at the Estação Ecológica de Seridó (ESEC–S, Seridó Ecological Station) (06°37′30″ S, 37°14′30″ W), in Rio Grande do Norte State [19]. The ESEC–S covers approximately 13 km^2^, with shrub and tree vegetation at its highest elevation (250–385 m). The annual average rainfall is 450 mm, and retention ponds in the park hold the water supply for the local population. The rural communities that surround the protected area practice agricultural activities. The samples were collected on two consecutive days per month over four months in the dry season and four in the wet season, from 2009 to 2010. Eggs were collected with ovitraps, and adults were collected during the day and at night with human landing catches and Shannon traps. Inácio [21] conducted the other survey in a private area of the Caatinga called Sítio Areias (SA), in the city of Currais Novos (06°16′50.60″ S, 36°28′23.70″ W), also in Rio Grande do Norte State. The area has tree–shrub vegetation known as the Caatinga hyperxerophilic seridó, with larger trees at higher altitudes. Tank bromeliads are rare all over the area. The annual precipitation is approximately 610 mm. The collections using Shannon traps and ovitraps were biweekly for three months in the dry period and another three months in the rainy season, in 2014 and 2015. Both studies are master’s dissertations and were published in collaboration with other studies in the Checklist of Mosquito of Rio Grande do Norte State by Inacio et al. [22].

Species names were abbreviated according to Reinert [26], following Harbach’s list of valid species [27]. For species of the genus *Aedes*, the old taxonomic classification was maintained, as recommended by Wilkerson et al. [28].

### 2.2. DNA Barcoding

Some species collected in the “Mosquitos da Caatinga” project had their CO1 gene studied for taxonomic purposes. Five representative specimens of each species of interest were processed. DNA was extracted from whole adult specimens and dried over silica gel, at −4 °C, using the DNeasy Blood & Tissue (Qiagen^®^, Valencia, CA, USA) kit. The products were stored at −20 °C in the Entomological Collection at the Federal University of Sergipe, in Aracaju (Brazil) prior to the amplification of a fragment of the Cytochrome c oxidase subunit 1 (CO1) gene. The primers LCO1490 and HCO2198 [29] were used to amplify a ~658 bp fragment, located at the 5’ end of the COI sequence, which was trimmed to 609 bps. Both the PCR and the sequencing reaction were run using the Big Dye PCR Direct (Applied Biosystems^®^, Foster City, CA, USA) kit, according to the manufacturer’s instructions. For the three *Wyemoyia* (*Phoniomyia*) morphotypes the ITS2 ribossomal fragments were amplified using 5.8SF5’ and 28SR5’ universal primers [30].

The PCR products were electrophoresed in 1.5% agarose gel stained with GelRed™ Nucleic Acid Gel stain (Biotium Inc, Hayward, CA, USA). The products were sequenced in both directions with the same set of primers and analyzed in an ABI 3130 DNA Analyser (PE Applied Biosystems, Warrington, UK). The samples were sequenced with bidirectional primers and the quality of the chromatograms was verified visually in Chromas v2.6.5 (Technelysium Pty Ltd, Brisbane, Australia). Homologies, insertions/deletions (indels), and frameshifts were identified in MUSCLE v3.8.31 [31]. The sequences, obtained in FASTA format, were aligned in ClustalW [32]. The sequence from each specimen was compared to barcode sequences from GenBank using ‘Blast’ and assigned to mosquito species in BOLD using the ‘Identification Request’ function. The CO1 sequences were deposited in the EMBL nucleotide sequence database. 

### 2.3. Data Analyses

Considering the differences between collection techniques, we performed qualitative analysis using the presence and absence of species in the six areas. The communities were classified according to the species and morphospecies richness.

The *betapart* package in the R [33] software was used for analysis. The Jaccard dissimilarity index (ßJAC) proposed by Baselga and Orme [34] was used to assess the partitioning of beta diversity. The partitioning was performed to determine which ecological mechanism, turnover (ßJTU) or nestedness (ßJNE), had the greatest influence on the changes in species composition in the six areas. The databases were converted to presence and absence matrices that were used for clustering. The species with uncertain identification in the studies, indicated as sp., were not considered. However, species assigned as undescribed were included, given the potential to represent the level of endemicity of an area. The cluster analysis was performed with the Jaccard index and the UPGMA (unweighted pair group method with arithmetic mean) coefficient [35] to represent the similarity between the six areas using PAST (Paleontological Statistics) package version 3.19 [36]. 

## 3. Results

From the data sets analyzed, 82 taxa in 14 genera were listed for mosquitoes of the Caatinga biome, 65 were valid species and 17 morphotypes, 12 of which are likely novel undescribed species (Table 2). The richness was 49 (60%) species for NMG; 33 (40%) for MONA–GA; 26 (32%) for ESEC–RC; 17 (21%) for FLONA–Açu; 13 (16%) for SA; and 11 (13%) for ESEC–S. Of the total, 35 species were shared among sites, whereas 47 occurred only in one of the six areas. The area with the highest percentage of exclusive species was NMG, containing 30% of the total species collected, followed by MONA–GA and ESEC–RC with 11% each, SA with 2%, and FLONA–Açu with 1%. The novel undescribed species were found only in MONA–GA and ESEC–RC. 

The following species are widespread and were found in three or more of the six areas: *Anopheles albitarsis* s.l.; *An. argyritarsis* s.l.; *An. triannulatus* s.l.; *Aedeomyia squamipennis*; *Aedes scapularis*; *Ae. taeniorhynchus*; *Ae. aegypti*; *Haemagogus spegazzinii*; *Psorophora ferox*; *Coquillettidia nigricans*; *Cq. venezuelensis*; *Mansonia humeralis*; *Ma. indubitans*; *Ma. titillans*; *Ma. wilsoni* and *Uranotaenia lowii*.

From the CO1 sequences analyzed, *Culex* (*Mcx.*) sp. near *xenophobus*, *Hg. leucocelaenus*, and *Cx.* Gr. *Pleuristriatus* sp.1 had two haplotypes each, while *Ae. fulvithorax* and *Ae. terrens* had three. All other species had only a single haplotype. This resulted in a total sample of 24 haplotypes, from the 17 species or morphotypes, with 24 nucleotide sequences being deposited in the GenBank (Table 2), besides three ITS2 nucleotide sequences from *Wy. (Pho)* spp. The species *Ae. scapularis*, *Cx. maxi*, *Cx*. *conservator, Ae. taeniorhynchus* and *Hg. leucocelaenus* had CO1 sequences deposited previously in the GenBank. *Aedes terrens*, *Ae. fulvithorax*, and *Ma. indubitans* were fully morphogically identified and had CO1 sequences deposited for the first time. 

It was not possible to identify the taxa listed as *Cx.* nr. (*And.*) sp. 1, *Cx.* (*Mcx.*) Gr. *Imitator* sp.1, *Cx.* (*Mcx.*) Gr. *Pleuristriatus* sp. 1, *Cx.* (*Mcx.*) sp. nr. *xenophobus*, *Runchomyia* sp. 1, *Toxorhynchites* sp. 1 and *Tx.* sp. 2, *Wyeomyia* sp. 1, *Wy.* (*Pho*) sp.1, *Wy.* (*Pho*) sp.2, *Wy.* (*Pho*) sp.3, to the species level based on their morphology. In addition, no similar CO1 or ITS2 sequences were found in the genetic databases. 

**Figure 2:**
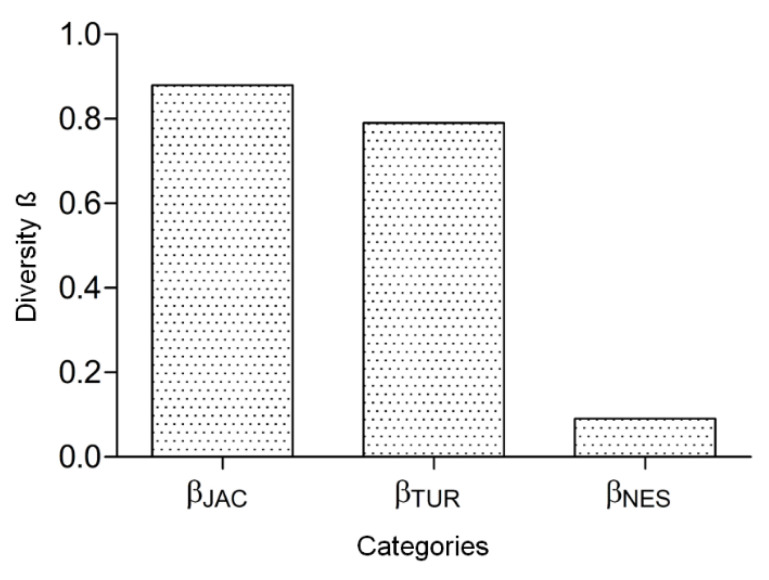
Mosquito beta diversity (ßJAC) and the partitioning of beta diversity into mechanisms of ecological turnover (ßJTU) and nestedness (ßJNE) among areas surveyed in the Caatinga biome between 2008 and 2014: MONA-GA—Grota do Angico Natural Monument in Sergipe; ESEC-RC—Raso da Catarina Ecological Station in Bahia; FLONA-Açu—Açu Natural Forest, ESEC-S—Seridó Ecological Station and SA—Sítio Areias in Rio Grande do Norte and NMG- North of Minas Gerais.

The ß diversity analysis showed a high total dissimilarity for species composition among the inventoried areas (ßJAC 0.88). The ecological process that best explained the species dissimilarity among mosquito communities at the regional scale was the spatial turnover of species (ßJTU 0.79), compared with nestedness or species loss (ßJNE 0.09) (Figure 2).

**Figure 3:**
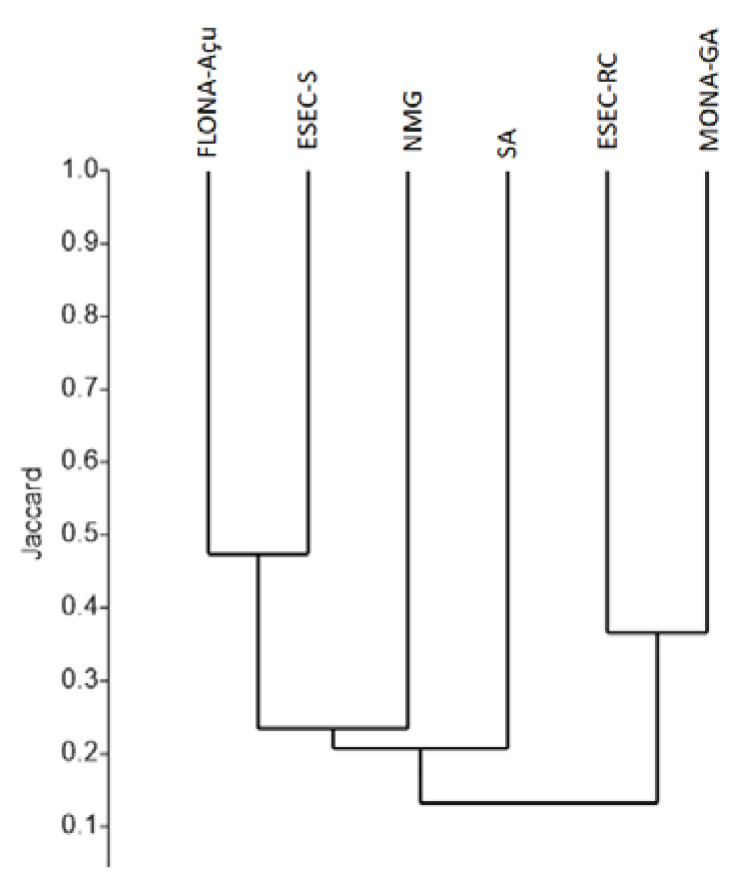
Clustering of the similarity (Jaccard dissimilarity index) of mosquito fauna in six areas of the Caatinga biome: MONA-GA—Grota do Angico Natural Monument in Sergipe; ESEC-RC—Raso da Catarina Ecological Station in Bahia; FLONA—Açu-Açu Natural Forest, ESEC-S—Seridó Ecological Station and SA—Sítio Areias in Rio Grande do Norte and NMG- North of Minas Gerais.

The cluster analysis showed similar species composition between FLONA–Açu and ESEC–S, and between ESEC–RC and MONA–GA, most likely because the sites of each pair are in close proximity. However, species composition in the protected areas in north of Minas Gerais and the areas surveyed in the state of Rio Grande do Norte (FLONA–Açu, ESEC–S, and SA) were more similar than those between ESEC–RC and MONA–GA, despite the distance, suggesting that other factors other than geographical proximity may influence the similarity (Figure 3).

## 4. Discussion

This study provides a recent picture of the diversity and spatial distribution of mosquito species in an environment with very limited aquatic habitats for larvae. Sixty-five mosquito species that were morphologically identified were distributed in the patches of dry deciduous forest areas. This number would be at least 82 considering the morphotypes that might represent novel undescribed species. Eighty-two species of mosquitoes is particularly impressive number as most of the surveys occurred during the largest drought of the last 40 years in the Brazilian semiarid region [11]. Moreover, the richness of mosquito species may be underestimated, since we excluded species recorded in the northeast region which could have not been caught in Caatinga. These species are *Aedes fluviatilis* (Lutz, 1904); *Anopheles aquasalis* Curry, 1932; *Anopheles fluminensis* Root, 1927; *Anopheles mediopunctatus* s.l. (Lutz, 1903)*; Anopheles oswaldoi* (Periassú, 1922); *Anopheles strodei* Root, 1926; *Anopheles nuneztovari* Gabaldon, 1940; *Culex taeniopus* Dyar & Knab, 1907; *Culex quinquefasciatus* Say, 1823 and *Toxorhynchites theobaldi* (Dyar & Knab 1906) [22,38]. 

The main limitation of this study is that the inventories to date have only surveyed a small portion of the total biome area. Sampled sites are of different sizes, ranging from 2.25 km^2^ (FLONA-Acu) to 1000 km^2^ (ESEC-RC) and the latter could have been under-sampled. The sampling period may also have affected the results unevenly, as some of them occurred during the period of extreme drought. In addition, not all surveys included larval habitats, which represent the best opportunity for collecting non-anthropophilic species that are usually not sampled by conventional adult mosquito capture methods. Species endemicity is also high in these habitats, as indicated by the number of novel undescribed mosquito species [9,10,11]. Therefore, the richness of mosquitoes might be higher than current knowledge suggests. Considering the mosquito species richness and endemicity, the hypothesis that the Caatinga is a homogeneous environment and poor in biodiversity can be rejected [39]. Future research should seek more representative and standardized sampling methods to better identify determinants of the spatial distribution of species.

The mosquito assemblage was more similar in closer areas, such as ESEC–S, FLONA–Açu, and SA and MONA–GA and ESEC–RC. However, at a regional scale, the more spatially distant areas, such as Rio Grande do Norte and the NMG, were more similar than sites separated by intermediate distances, such as MONA–GA and ESEC–RC. Sampling bias should be considered, since the similarity observed in the species clustering among sites coincides with the type of collection techniques employed (Table 1). Similarly, the local diversity was greater where there was a larger sampling effort, such as NMG and MONA–GA. Habitat specificity is likely important in the structuring of mosquito communities, considering the ecological interactions that regulate the local composition of a community. The habitat specificity of species could explain the similarity in mosquito communities between areas that are as far apart as NMG and the state of Rio Grande do Norte (RN). For example, *Mansonia* species were mostly shared between NMG and RN areas, where their ecological interaction with aquatic plants during immature phases [40] depends on the presence of ponds, that are virtually absent in the study area of ESEC-RC.

Although different factors may influence the formation of assemblages in different areas, the partitioning of beta diversity indicates that spatial turnover is the predominant ecological mechanism affecting the species composition of mosquito communities. The importance of species turnover may be due to the high environmental heterogeneity in the Caatinga phytogeographic domain, which increases the different niche conformations available to species [3,8,13,41]. That would explain the dominance by some endemic mosquito species [10,11]. Thus, spatial variation within this biome is of particular importance for species richness, and the deforestation of a relatively small area could lead to the extinction of certain species.

The mosquito species inhabiting the adverse environment of the Caatinga have developed evolutionary mechanisms to cope with drought and to maintain their populations until the rainy season. It has been verified for some species the necessity of several egg immersions and diapause [11,42,43]. These drought coping mechanisms may be responsible for the ecological mechanism of nesting, which was also observed in the present study, although nesting was less remarkable than the spatial turnover of species. The co-occurrence of species between communities reduces gamma diversity but reflects the tolerance of these species to survive under different environmental conditions. 

Abrupt environmental changes, such as flooding and deforestation, also contribute to variations in the abundance of mosquito populations [44]. Such changes could explain the high dissimilarity in species composition between communities. From an epidemiological perspective, environmental degradation interferes in the dynamics of vector borne diseases as when the mosquitoes’ natural habitats are destroyed, the proliferation of anthropophilic species is favored. Thus, high diversity can help to protect people from mosquito-transmitted diseases [45].

Most of the species listed in Caatinga biome would be found from north to south of Brazil, in the dry or the rain forests [46,47,48]. Even though some captured in just one of the six areas, as *An. marajoara*, *Hg. janthinomys*, *Ae. fulvus* and others, can be found in other biomes in Brazil, some species are more specialized and endemic. The molecular study showed a relevant number of undescribed species among those to whom the morphological identification was not possible. *Culex (Mcx.)* sp. nr. *xenophobus* CO1 are similar to those of the other members of the *Microculex* subgenus, such as *Cx.* Gr. *Imitator* sp. 1 and *Cx.* Gr. *Pleuristriatus* sp. 1, despite being separated by a divergence of approximately 12%. The genetic divergence between the MONA-GA and ESEC-RC haplotypes was greater than 2%. Clearly, then, these two haplotypes require further taxonomic analysis and may indicate a process of allopatric speciation.

A CO1 sequence of *Cx. conservator* was found in the database (KF671017), although the pairwise comparison with our sequence revealed a divergence of 8%. A more detailed reading of Linton et al. [49] revealed that sequence KF671017 was identified as *Culex* sp. near *conservator*, what may justify the divergency with the one collected in Caatinga. However, our sequences of *Cx. conservator* diverged by only 0.2% from the database sequence of *Culex browni* Kompi, 1936 (KF671016), with a difference of only two nucleotides. Even so, our species is morphologically distinct from *Cx. browni*, based on the dichotomous identification key of Berlin and Belkin [50], given the short gills of the larvae, the creamy erect scales on the vertex of the adults, and the sub-basal leaf on the distal division of the sub-apical lobe of the male genitalia. This set of characters is sufficient to distinguish the two species morphologically. The lack of CO1 barcode sequences in the databases makes it difficult to analyze the distribution of base substitution events that may contribute to a more precise taxonomic identification. Either way, this phytotelmata mosquito subgenus seems to be adapted to the conditions of the Caatinga, with several species, and some of which are likely endemic of this biome. 

## 5. Conclusions

The diversity of mosquitoes is considerable in the Caatinga, with 65 valid species and 17 morphotypes of which at least 12 were exhaustively studied and are unknown species to be described. Additionally, this diversity was determined based on the few surveys available. The determining factors of mosquito community are the wide environmental heterogeneity, the intrinsic relationships of the species with their habitats, and the environmental degradation caused by different types of anthropogenic interference. This result reinforces the need for a deeper understanding of the mosquito diversity in Brazil beyond the study of species of epidemiological interest, including the description of the new species that were found. 

The northeast region of Brazil is widely populated and subjected to landscape changes, whether clandestine activities such as deforestation and extractivism or initiatives for development such as irrigation projects, settlements, transposition of rivers, and dams. The consequences of these activities for changes in the composition of mosquito communities may emerge in the near future. The results obtained in this work are also expected to contribute to the elaboration of contingency plans for emerging and re-emerging tropical diseases in the Caatinga as a result of these anthropogenic alterations.

## Figures and Tables

**Table 1:** Set of collection techniques applied and habitats searched in surveys conducted in the Caatinga biome between 2008 and 2015.

Techniqueand Habitats	Survey Areas * and Time					
	MONA–GA 2011–2014	ESEC–RC 2013–2014	FLONA–Açu 2011–2013	ESEC–S 2009–2010	SA/RN 2014–2015	NMG 2008–2012
Human landing catches	x	x	-	x	-	x
Shannon trap	x	x	x	x	x	x
Tree holes	x	x	-	-	-	-
Bromeliads	x	x	-	-	-	-
Ponds	x	x	-	-	-	-
Larvitrap	-	-	x	-	-	-
Ovitrap	x	-	x	x	x	-
CDC light trap	-	-	-	-	-	x

x = Technique was used in the study; - Technique not used.* MONA–GA/SE, Monumento Natural Grota do Angico in Sergipe; ESEC–RC/BA, Estação Ecológica Raso da Catarina in Bahia; FLONA–Açu/RN, Floresta Natural do Açu in Rio Grande do Norte; ESEC–S/RN, Estação Ecológica do Seridó in Rio Grande do Norte; SA/RN, Sítio Areias in Rio Grande do Norte; NMG, north of Minas Gerais.

**Table 2:** Checklist of the regional diversity of Culicidae gathered from surveys conducted in protected areas in the Caatinga biome between 2008 and 2015.

N	Species	Collections Sites ^a^						CO1 Access Number
		MONAGA /SE	ESEC RC/BA	FLONA /RN	ESEC S/RN	SA RN	NMG	
	**ANOPHELINAE**							
1	*Anopheles albitarsis* s.l. Lynch Arribalzaga, 1878	x	x	x	x	x	x	
^b^	*An. argyritarsis* s.l. Robineau-Desvoidy, 1827	x	x				x	
2	*An. argyritarsis* s.s. Robineau-Desvoidy, 1827	x						KT762353 ^d^
3	*An. braziliensis* (Chagas, 1907)					x	x	
4	*An. darlingi* Root, 1926						x	
5	*An. deaneorum* Rosa-Freitas, 1989					x	x	
6	*An. evansae* (Brèthes, 1926)						x	
7	*An. marajoara* Galvão & Damasceno, 1942					x		
8	*An. oryzalimnetes* Wilkerson & Motoki, 2009	x ^c^						KT762355 ^d^
9	*An. sawyeri* Causey, Deane, Deane & Sampaio, 1943	x ^c^						KT762356 ^d^
10	*An. albimanus* section/Oswaldoi Subgroup						x	
11	*An. triannulatus* s.l. (Neiva & Pinto, 1922)	x	x		x		x	
	**CULICINAE**							
	**Aedomyiini**							
12	*Aedeomyia squamipennis* (Lynch Arribalzaga, 1878)	x		x	x		x	
	**Aedini**							
13	*Aedes fulvithorax* (Lutz, 1904)		x ^c^				x	MH118159 MH118160 MH118161
14	*Ae. fulvus* (Wiedemann, 1828)						x	
15	*Ae. hastatus* (Dyar, 1922)						x	
16	*Ae. lepidus* (Cerqueira & Paraense, 1945)					x		
17	*Ae. scapularis* (Rondani,1848)	x	x ^c^	x	x	x	x	MH118146
18	*Ae. serratus* (Theobald, 1901)						x	
19	*Ae. stigmaticus* (Edwards, 1922)						x	
20	*Ae. taeniorhynchus* (Wiedemann, 1821)	x ^c^		x		x	x	MH118152
21	*Ae. terrens* (Walker, 1856)	x	x ^c^					MH118164 MH118165 MH118166
22	*Ae. aegypti* (Linnaeus, 1762)			x	x		x	
23	*Ae. albopictus* (Skuse, 1895)			x				
24	*Haemagogus janthinomys* Dyar, 1921						x	
*25*	*Hg. spegazzinii* Brethés, 1912			x		x	x	
26	*Hg.* sp. near *spegazzinii* Brethés, 1912	x ^c^	x					MH118155
27	*Hg. leucocelaenus* (Dyar & Shannon, 1924)		x ^c^				x	MH118162 MH118163
28	*Psorophora cingulata* (Fabricius, 1805)						x	
29	*Ps. discrucians* (Walker, 1856)						x	
30	*Ps. ferox* (Von Humboldt, 1819)	x			x	x	x	
31	*Ps. albigenu* (Peryassú, 1908)						x	
	**Mansoniini**							
*32*	*Coquillettidia shannoni* (Lane & Antunes, 1937)	x						
*33*	*Cq. albicosta* (Peryassú 1908)						x	
34	*Cq. hermanoi* (Lane & Coutinho, 1940)						x	
35	*Cq. juxtamansonia* (Chagas, 1907)			x			x	
36	*Cq. lynchi* (Shannon, 1931)						x	
37	*Cq. nigricans* (Coquillett, 1904)			x	x		x	
38	*Cq. venezuelensis* (Theobald, 1912)			x	x		x	
39	*Mansonia humeralis* Dyar & Knab, 1916			x	x		x	
40	*Ma. indubitans* Dyar & Shannon, 1925	x ^c^	x ^c^	x		x	x	MH118158
*41*	*Ma. pseudotitillans* (Theobald, 1901)			x			x	
*42*	*Ma. titillans* (Walker, 1848)			x	x		x	
*43*	*Ma. wilsoni* (Barreto & Coutinho, 1944)			x	x	x		
	**Culicini**							
44	*Culex conservator* Dyar & Knab 1906	x ^c^	x					MH118148
45	*Cx.* (*And*.) sp. 1		x					
46	*Culex* nr. (*And*.) sp. 1	x ^c^						MH118147
47	*Cx. ameliae* Casal, 1967						x	
48	*Cx. bidens* Dyar, 1922						x	
49	*Cx. chidesteri* Dyar, 1921	x		x				
50	*Cx.* Group *coronator* Dyar & Knab, 1906	x				x		
51	*Cx. declarator* Dyar & Knab, 1906	x						
52	*Cx. habilitator* Dyar & Knab, 1906	x					x	
53	*Cx. maxi* Dyar, 1928	x ^c^	x					MH118167
54	*Cx. nigripalpus* Theobald, 1901	x	x					
55	*Cx. restuans* Theobald, 1901						x	
56	*Cx. salinarius* Coquillett, 1904						x	
57	*Cx. saltanensis* Dyar, 1928	x	x				x	
*58*	*Cx. scimitar* Branch & Seabrook, 1959						x	
*59*	*Cx. imitator* Theobald, 1903		x					
*60*	*Cx.* Gr. *Imitator* sp. 1 Theobald, 1903		x ^c^					MH118149
*61*	*Cx.* Gr. *Pleuristriatus* sp. 1 Theobald, 1903		x ^c^					MH118150 MH118151
*62*	*Cx.* sp. nr. *xenophobus*	x ^c^	x					MH118153 MH118154
*63*	*Cx. aureonotatus* Duret & Barreto, 1956	x						
*64*	*Cx. bastagarius* Dyar & Knab, 1906	x						
65	*Cx.* Complex *Vomerifer*						x	
66	*Cx.* Group *Atratus*						x	
67	*Cx. ribeirensis/cedecei*	x						
68	*Cx.* Section *melanoconion*			x			x	
	**Sabethini**							
69	*Limatus durhamii* Theobald, 1901	x						
70	*Li. paraensis* (Theobald, 1903)						x	
71	*Runchomyia* sp. 1		x ^c^					MH118156
72	*Sabethes undosus* (Coquillett, 1906)						x	
73	*Wyeomyia* (*Phoniomyia*) sp. 1	x	x ^c^					MT152293 ^e^
*74*	*Wy.* (*Pho.*) sp. 2	x	x ^c^					MT152294 ^e^
*75*	*Wy.* (*Pho.*) sp. 3		x ^c^					MT152295 ^e^
*76*	*Wy.* sp. 1		x ^c^					MH118157
	**Toxorhynchitini**							
77	*Toxorhynchites* (*Lynchiella*) sp. 1	x	x ^c^					MF537258
78	*Tx.* (*Lyn.*) sp. 2	x	x ^c^					MF537259
	**Uranotaeniini**							
*79*	*Uranotaenia geometrica* Theobald, 1901						x	
80	*Ur. lowii* Theobald, 1901	x	x			x	x	
81	*Ur. pulcherrima* Lynch Arribalzaga, 1891						x	
82	*Ur. apicalis Theobald, 1903*	x						
	**Total**	33	26	13	11	13	49	

x = presence; blank space = absence. ^a^ MONA–GA/SE, Monumento Natural Grota do Angico in Sergipe; ESEC–RC/BA, Estação Ecológica Raso da Catarina in Bahia; FLONA–Açu/RN, Floresta Natural do Açu in Rio Grande do Norte; ESEC–S/RN, Estação Ecológica do Seridó in Rio Grande do Norte; SA/RN, Sítio Areias in Rio Grande do Norte; NMG, north of Minas Gerais. ^b^ Referes to *An. argyritarsis* s.s. or *An. sawyeri* already included in the list. ^c^ Sample of CO1 deposited in GenBank. ^d^ Published by Marteis et. al. [37]. ^e^ ITS2.
